# Integrative analysis of DNA methylation and gene expression profiles identified potential breast cancer-specific diagnostic markers

**DOI:** 10.1042/BSR20201053

**Published:** 2020-05-27

**Authors:** Xinhua Liu, Yonglin Peng, Ju Wang

**Affiliations:** School of Biomedical Engineering, Tianjin Medical University, Tianjin 300070, China

**Keywords:** ATAC-seq, breast cancer, ChIP-seq, diagnostic, logistic regression, TCGA

## Abstract

Breast cancer is a common malignant tumor among women whose prognosis is largely determined by the period and accuracy of diagnosis. We here propose to identify a robust DNA methylation-based breast cancer-specific diagnostic signature. Genome-wide DNA methylation and gene expression profiles of breast cancer patients along with their adjacent normal tissues from the Cancer Genome Atlas (TCGA) were obtained as the training set. CpGs that with significantly elevated methylation level in breast cancer than not only their adjacent normal tissues and the other ten common cancers from TCGA but also the healthy breast tissues from the Gene Expression Omnibus (GEO) were finally remained for logistic regression analysis. Another independent breast cancer DNA methylation dataset from GEO was used as the testing set. Lots of CpGs were hyper-methylated in breast cancer samples compared with adjacent normal tissues, which tend to be negatively correlated with gene expressions. Eight CpGs located at RIIAD1, ENPP2, ESPN, and ETS1, were finally retained. The diagnostic model was reliable in separating BRCA from normal samples. Besides, chromatin accessibility status of RIIAD1, ENPP2, ESPN and ETS1 showed great differences between MCF-7 and MDA-MB-231 cell lines. In conclusion, the present study should be helpful for breast cancer early and accurate diagnosis.

## Introduction

Breast cancer (BRCA) is one of the most common cancers and the second cause of cancer-related death among women [1]. Lots of risk factors, including environmental and genetic aspects, have been increasingly revealed to be carcinogenic in BRCA. Although remarkable advancement in BRCA care in recent years, taking genomic information into account in personal diagnosis is still urgently needed in precision medicine for improving patients’ prognosis [2,3].

Imaging techniques, including mammography, computed tomography, magnetic resonance imaging, and so on, have been used as the primary means for BRCA diagnosis and patients monitoring until now [4,5]. Several biochemical-based methods have also been developed for overcoming potential limitations of imaging methods, such as high spending and low sensitivity or specificity in special cases [6]. Lots of BRCA-specific biochemical biomarkers, in addition to the pan-cancer markers, have been identified and proved their potential applications in BRCA diagnosis and stage monitoring. For example, BRCA1/2 germline mutations, which are widely considered to cause DNA damage repair (DDR) defection, have been extensively applied in BRCA diagnosis and early screening, as well as the subsequent treatment decision making worldwide [7–9]. MicroRNA, a type of regulatory small molecule that without protein-coding potential, which plays pivotal roles in post-transcriptional gene expression regulations, is emerging as not only BRCA diagnostic but also prognostic biomarkers [10–13]. Besides, combination of imaging techniques and biomedical biomarkers are also recommended for further improving BRCA diagnosis and staging [14].

DNA methylation (DNAme) represents the most common DNA epigenetic modification that mostly occurs at the 3-cytosine of CG dinucleotide (CpG) and plays a vital role in gene regulation. Specifically, hyper-methylation particularly those located at cis-regulation element (CRE), including enhancer and promoter, could hinder the binding of transcription factor and hence cause gene transcription silencing, which is considered as the basic mechanism of carcinogenesis [15–17]. What’s more, DNA methylation as the most common epigenetics modification could contribute lots of other biological processes, such as cell division [18,19] and stem cell differentiation [20,21], which could consequently influence physical activities, such as aging [22], tumorigenesis [23], metabolism [24,25], and so on. DNAme has always been applied in early diagnosis of multiple cancers including BRCA for its prevalence in cancer cells and stable inheritance from parent to daughter cells. For example, Harri et al. [26] identified a specific DNA region named EFC#93 whose serum methylation pattern might serve as an early diagnostic marker of disseminated BRCA. Esteller et al. [27] developed a DNAme-based signature by which BRCA could be definitely divided into two subtypes that with significantly different clinicopathological features and prognosis. While majority of those studies are mainly focused on comparison between BRCA and normal samples or intra-BRCA samples, and seldomly on DNAme specificity of BRCA compared with other common cancers, which should be helpful in BRCA diagnostic sensitivity and specificity.

In the present study, we comprehensively investigated the genome-wide DNAme landscape of BRCA and other 10 cancers and their adjacent normal tissues and healthy breast tissues from the Cancer Genome Atlas (TCGA) and Gene Expression Omnibus (GEO). Eight BRCA-specific CpGs were finally obtained and a logistic regression model was constructed through which BRCA could be distinguished from normal samples and other cancers with high sensitivity and specificity.

## Materials and methods

### Study population

All the subjects in the present study were obtained from publicly accessible databases, i.e. the Cancer Genome Atlas (TCGA) and Gene Expression Omnibus (GEO). Training set, which was used for screening breast cancer (BRCA)-specific diagnostic markers, is composed of tumor and adjacent normal tissues of BRCA and other 10 cancers, including BLCA, COAD, GBM, HNSCC, KIRC, LIHC, LUAD, LUSC, READ, and UCEC (full names of those abbreviations could be accessed from https://gdc.cancer.gov/resources-tcga-users/tcga-code-tables/tcga-study-abbreviations) in TCGA. Numbers of tumor and adjacent normal samples of those cancers were provided in Table 1. Besides, GSE88883 [28] from GEO, containing genome-wide methylation profiles of 100 healthy breast biopsies, was also included in the training set. GSE66695, including 40 normal and 80 BRCA samples, was used as testing set for evaluating performance of BRCA-specific diagnostic markers.

**Table 1:** Numbers of tumor and paracancerous tissues contained in every TCGA dataset that used in the present study

Dataset	Tumor tissue	Paracancerous tissue
BRCA	783	109
BLCA	419	21
COAD	315	38
GBM	153	2
HNSCC	530	50
KIRC	325	160
LIHC	380	50
LUAD	475	32
LUSC	370	42
READ	99	7
UCEC	439	46

### Differential methylation and expression analysis

Genome-wide methylation profiles of samples in both training and testing set were detected by using Infinium Human Methylation 450K BeadChip (Illumina, Inc, San Diego), which covers 96% CpG island (CGI) of human genome and 99% RefSeq genes with more than 480,000 probes. CpGs locate at sex chromosomes and those contain SNPs were first removed from the subsequent differential methylation analysis. We also excluded CpGs with detection *P* value > 0.01 in more than half of all samples. Methylation levels, which were also denoted as β values, of the remaining CpGs were calculated as the ratio of methylated probe intensity and the sum of methylated and unmethylated probe intensity, which range from 0 to 1. Normality of β values across samples was determined by Kolmogorov–Smirnov (K-S) test for the proper selection of statistical method in differential methylation analysis. Comparisons between paired tumor and adjacent normal samples and between unpaired tumor and normal samples were performed by using paired and unpaired *t*-test (normal distribution) or Wilcoxon signed-rank test (abnormal distribution), respectively. Absolute delta β value > 0.2 and FDR adjusted *P* value < 0.01 was used as the threshold for identification of differential methylation CpGs (DMCs).

Level3 RNA-seq datasets, i.e. raw count of reads mapped to each gene, of TCGA-BRCA project were used for the identification of differential expression genes (DEGs) in BRCA compared with adjacent normal samples via DESeq2 Bioconductor package. Genes satisfied the threshold of absolute log2-based fold change (FC) > 1 and FDR adjusted *P* value < 0.01 were considered as significantly differential expression.

### CpG island methylator phenotype identification

CpG island methylator phenotype (CIMP) represent an indicator for estimating the overall methylation level in genes’ promoters for a specific sample, which has exhibited great prognostic value in multiple cancers. Here, we proposed to define CIMP for BRCA samples based on their DMCs’ methylation levels. Sample clustering analysis based on CpGs’ methylation levels was carried out through *K*-means method, then samples in the cluster that with highest methylation levels were defined as CIMP samples and the remaining samples were defined as non-CIMP samples.

### Cis- and trans-regulation analysis

Aberrant methylation of promoter CpGs may induce the altered expression of the corresponding gene through cleaving transcription factor from oligonucleotides, and this process is called as cis-regulation between CpG and gene. CpGs could also influence expression of genes that far away from them in genome position if their locations are happened to be the important regulatory elements of those genes, such as enhancer, and this type of regulation between CpG and gene is referred as trans-regulation. In the present study, we explored both cis- and trans-regulation relationship between DMCs and DEGs by calculating Pearson correlation coefficients between CpG’s β value and gene expression. *P* value < 0.05 was used as the threshold for correlation significance.

### Logistic regression analysis and performance evaluation

DMCs with significantly elevated methylation in BRCA tumor tissues than adjacent normal tissues in paired as well as unpaired comparisons were retained for further screening of CpGs that showed significantly hyper-methylation in BRCA tumor samples compared with any of the other ten cancers’ tumor and adjacent normal tissues as well as healthy breast tissues. The finally remained CpGs were defined as BRCA-specific CpGs and were applied in logistic regression analysis. Sample type of samples in the training set, i.e. BRCA or adjacent normal sample, and CpGs’ β values, were used as categorical responsive and continuous predictive variables, respectively, in the logistic regression analysis. Samples in the testing set were predicted for their type by using the logistic regression model. Receiver operating characteristic (ROC) curve was plotted and area under curve (AUC) was used for evaluating the sensitivity and specificity of the model in BRCA diagnosis.

### ATAC-seq and ChIP-seq analysis

In some genes, CpGs within CGIs that located at promoter could directly occupy unstable nucleosomes and thereby influence nucleosomes packing, i.e. chromatin accessibility in other words [29]. Here, we proposed to explore if genes that contain BRCA-specific CpGs exhibited overall different chromatin accessibility landscape in BRCA cell lines that have variable aggressive degree by analyzing Assay for Transposase-Accessible Chromatin using sequencing (ATAC-seq) and H3K27ac Chromatin Immunoprecipitation coupled with sequencing (ChIP-seq) datasets in MCF-7 and MDA-MB-231 cell lines. Raw sequencing data were obtained from GEO with the following accession numbers: GSM2714245 [30] (ATAC-seq, MCF-7), GSM2067520 (H3K27ac ChIP-seq, MCF-7), GSM1855976/GSM1855977/GSM1855978 [31] (ATAC-seq rep1/rep2/rep3, MDA-MB-231), GSM1855991/GSM1855992 [31] (H3K27ac ChIP-seq rep1/rep2, MDA-MB-231). Removal of sequencing adapters, low-quality bases and reads, and unpaired reads was first performed by using fastp [32], an ultra-fast all-in-one fastq preprocessor, with the default parameters setting. Bowtie2 [33], with the maximal gap (-X) between two paired reads as 2000 and keep ‘–very-sensitive’ option on, was used for the alignment of preprocessed reads to hg38/GRCh38 reference genome. Mapping files in sam format were converted to files in bam format by using samtools version1.5 [34] followed by bamCoverage application of deepTools version2.0 [35] for calculating reads intensity across the genome. Integrative Genomics Viewer (IGV) [36] was used to visualize sequencing read intensity profiles at specific region.

### Statistical analysis

Significance of difference of specific gene’s expression values between adjacent normal and tumor tissues was determined by paired *t*-test. Overall survival (OS) probabilities of BRCA patients were estimated through Kaplan–Meier method, which were then compared among different groups by using log-rank test. Fisher’s exact test was used to elevating genome distribution’s difference between hyper- and hypo-methylation CpGs. All the statistical analyses were performed in R version3.4.1. *P* value < 0.01 or 0.05 was used as the significant threshold.

## Results

### CpG island methylator phenotype identification

**Figure 1:**
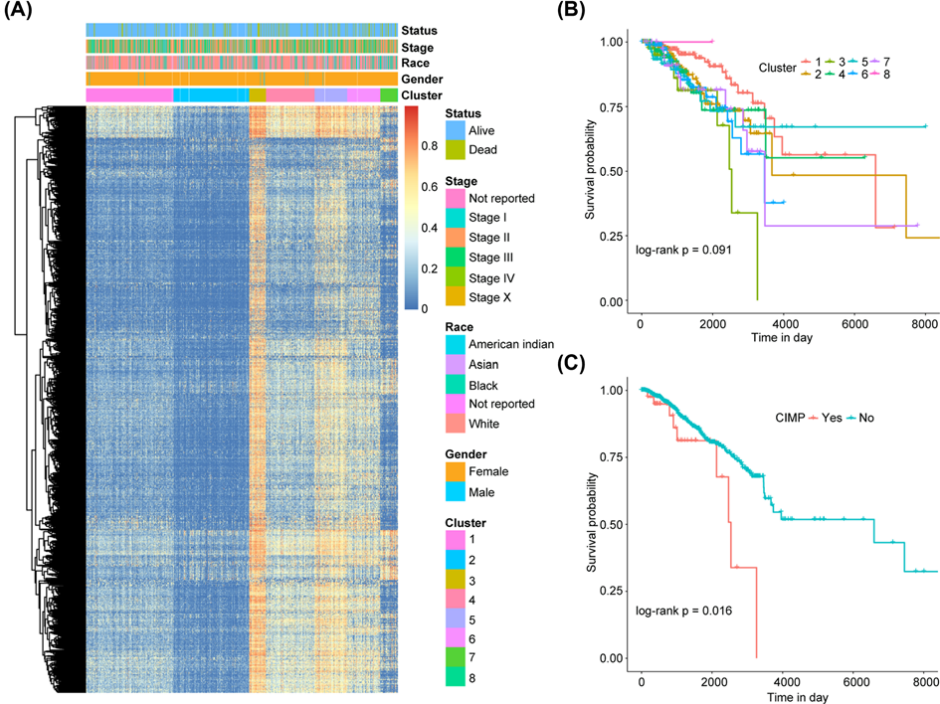
Methylation landscape of BRCA samples and association between CIMP and patients’ overall survival (**A**) Heatmap depicts clustering of BRCA samples (column) based on CpGs (row) with sd of β value greater than 0.2 among all BRCA samples and mean β value of adjacent normal tissues less than 0.05 through *K*-means method. Epidemiology information of BRCA patients including living status, stage, race, and gender besides their cluster information were provided at the top of the heatmap and differentiated with colors. (**B**) Kaplan–Meier curves of BRCA samples stratified by their cluster information. Horizontal and vertical axis represents patients’ overall survival in days and survival probability, respectively. *P* value was determined by log-rank test. (**C**) Kaplan–Meier curves of BRCA samples stratified by CIMP status. Horizontal and vertical axis represents patients’ overall survival in days and survival probability, respectively. *P* value was determined by log-rank test.

Preprocessing of raw TCGA-BRCA methylation datasets retained a total of 343,451 CpGs that located at autosomes and with low detection *P* value (<0.01) across more than half of samples. CpG island methylator phenotype (CIMP), which is used for describing CpG island promoter methylation of specific tumors and closely correlated with tumorigenesis and prognosis, was initially developed in colorectal cancer and has been extremely applied in many other cancers including BRCA. Here, we proposed to identify BRCA samples with CIMP through clustering analysis. We first identified a total of 2959 CpGs whose β value’s standard error (sd) > 0.2 among all the 783 BRCA tumor samples and mean β value of the 109 adjacent normal samples < 0.05. *K*-means clustering of BRCA tumor samples was performed based on their Euclidean distances, which were calculated via the 2,959 CpGs, with the optimal cluster number of 8 determined by consensus clustering analysis (Supplementary Figure S1). As shown in Figure 1A, cluster3, which contains 42 BRCA samples, has higher overall methylation levels across the 2959 CpGs than other seven clusters and thus was defined as CIMP cluster. Log-rank test indicated a marginal significance (*P* value = 0.091) of OS probability difference among all the 8 clusters (Figure 1B). While, significantly inferior relationship between CIMP and BRCA patients’ OS probability was obtained when grouped tumor samples according to their CIMP status, i.e. with (cluster3) and without (other seven clusters) CIMP as shown in Figure 1C.

### Hyper-methylation CpGs tend to locate at cis-regulation element

**Figure 2:**
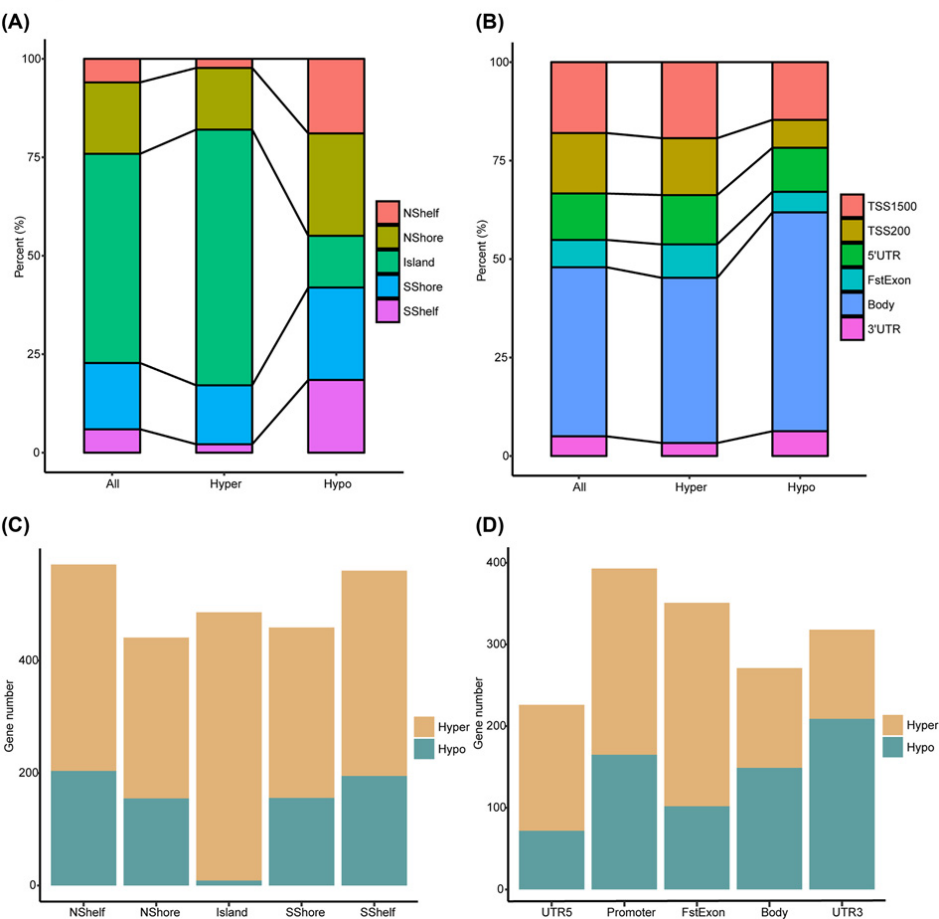
Site- and region-level differential methylation analysis (**A** and **B**) Distribution of all retained CpGs in the microarray, hyper- and hypo-methylation CpGs across different genome features that defined by the distance relative to CpG island and transcription start site. (**C** and **D**) Stack graph depicts number of genes exhibit hyper- and hypo-methylation at different genome regions defined by the distance relative to CpG island and transcription start site.

K–S test indicated normality of CpGs’ β value distribution across all the samples (data not shown). Paired differential methylation analysis identified 29,762 DMCs in tumor samples compared with adjacent normal samples through paired *t*-test, and 26,418 CpGs were still significant in unpaired methylation comparison, i.e. all BRCA tumor samples versus adjacent normal samples, which including 15,415 hyper-methylation CpGs (hyper-CpGs) and 11,003 hypo-methylation CpGs (hypo-CpGs). Hyper-, hypo-CpGs, as well as all the 343,451 CpGs were analyzed for their distributions across genome features relative to CGI, including North Shelf (NShelf, 2000–4000 bp upstream of CGI), North Shore (NShore, 0–2000 bp upstream of CGI), CGI, South Shore (SShore, 0–2000 bp downstream of CGI), and South Shelf (SShelf, 2000–4000 bp downstream of CGI) (Figure 2A). Distributions of those CpGs across genome features relative to transcriptional start site (TSS), including TSS1500 (200–1500 bp upstream of TSS), TSS200 (0–200 bp upstream of TSS), 5′UTR, first exon (FstExon), gene body, and 3′UTR, are provided in Figure 2B. Vital roles of elevated CGI methylation level in promoter have been previously highlighted in carcinogenesis, which was also well characterized in the present study. Specifically, hyper-CpGs were more prone to locate at CGI than hypo-CpGs (Figure 2A, Fisher’s exact test, *P* value = 0, odd ratio (OR) = 24.54, 95% confidence interval (CI): 22.29–27.89), as well as all CpGs (Figure 2A, Fisher’s exact test, *P* value = 0, OR = 1.90, 95% CI: 1.85–1.96), and were more prone to locate at promoter, i.e. TSS200 and TSS1500, than hypo-CpGs (Figure 2B, Fisher’s exact test, *P* value = 1.958 × 10^−42^, OR = 1.55, 95% CI: 1.46–1.66).

Region-level aberrant methylation was previously proven to be closely associated with complex disease progression by mechanistically regulating gene expression and cell differentiation. Here, we divided a specific gene into multiple regions, including CGI, NShore, NShelf, SShore, and SShelf according to CGI, or TSS200, TSS1500, 5′UTR, FstExon, Body, and 3′UTR according to TSS, and summarized the region-level methylation as mean methylation value of CpGs contained in the corresponding region. Comparison of region-level methylation between paired adjacent normal and tumor samples was performed by paired *t*-test, and the results indicated distinct higher number of genes that exhibited elevated methylation than those exhibited decreased methylation in tumor samples in CGI region as shown in Figure 2C. Besides, genes were more frequently hyper-methylated at the upstream of the TSS, including TSS200, TSS1500, and FstExon, which were more likely CREs, and hypo-methylated at the downstream of the TSS in our study as shown in Figure 2D.

### Hyper-CpGs tend to negatively regulate gene expressions

**Figure 3:**
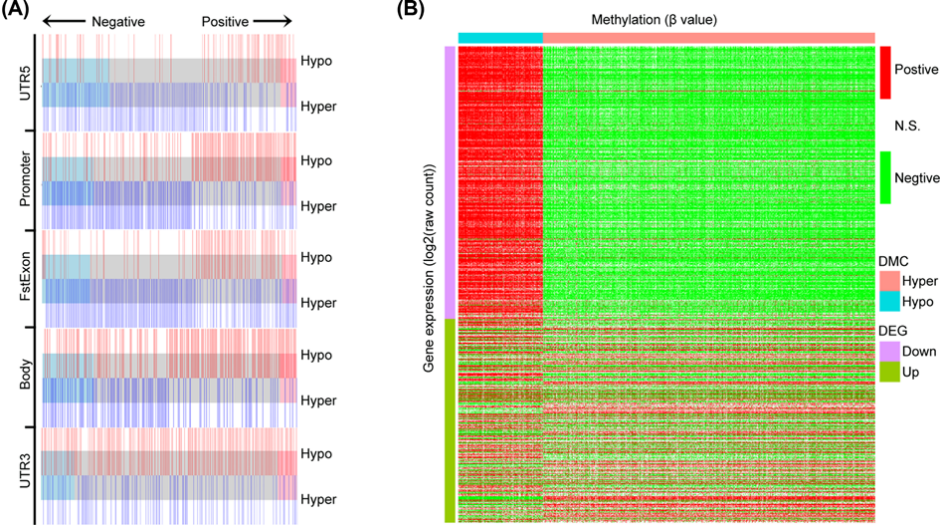
Cis- and trans-regulation analysis based on CpGs’ β value and mRNA expression (**A**) Enrichment plot illustrates cis-regulation relationship between hyper-/hypo-methylation CpGs and their corresponding genes across different genome regions defined by the distance relative to transcription start site. A line in the plot indicates one correlation coefficient. Neg, negative correlation; Pos, positive correlation. (**B**) Heatmap depicts trans-regulation relationship between hyper-/hypo-methylation CpGs that located in DEGs and DEGs. Neg, negative correlation; Pos, positive correlation; NS, not significant.

Hyper-CpGs, particularly those located at CREs, such as promoter and enhancer, were cognitively considered to suppress gene expressions in cis- or trans-regulation pattern. We here first explored cis-regulation correlations of hyper- and hypo-CpGs with their genes stratified by their genome locations relative to TSS through calculating Pearson correlation coefficients between CpGs’ β values and gene expressions. Strikingly, hyper- and hypo-CpGs were substantially negatively and positively correlated with their gene expressions, respectively, across all the genome locations except 3′UTR, in which correlations between DMCs and their genes were evenly distributed across negative and positive conditions (Figure 3A). In trans-regulations, we mainly focused on those correlations between DMCs and DEGs, which including 3486 up- and 2437 down-regulated ones in BRCA tumor samples, and similar results were obtained as cis-regulation analysis. Specifically, by dividing DMCs into hyper- and hypo-CpGs, and DEGs into up- and down-regulated genes, extreme biases toward negative/positive correlation between hyper-/hypo-CpGs and down-regulated genes could be observed as shown in Figure 3B. In view of the epigenetics mechanism of tumorigenesis, our data should implicate pivotal roles of the two regulation patterns, through which the hyper-CpGs could silence tumor suppressor genes.

### BRCA-specific diagnostic CpGs

**Figure 4:**
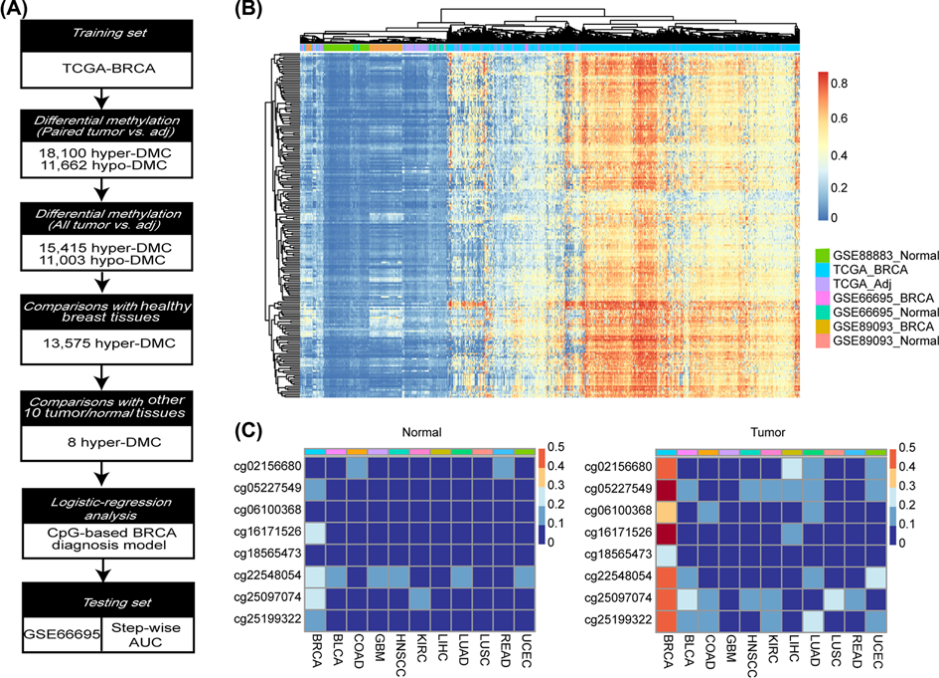
Identification of BRCA-specific diagnostic CpGs (**A**) Workflow for screening BRCA-specific diagnostic CpGs. (**B**) Heatmap illustrates clustering of BRCA and normal samples based on the top 200 CpGs exhibit most significant differential methylation in the comparison between BRCA and normal samples from TCGA as well as GEO. (**C**) Heatmap for the methylation level of the eight BRCA-specific diagnostic CpGs across the normal (left panel) and tumor tissues (right panel) of other ten cancers besides BRCA from TCGA.

Figure 4A illustrates the workflow for BRCA-specific diagnostic CpGs identification and evaluation. Following differential methylation analysis, the 15,415 hyper-CpGs were further compared between the TCGA-BRCA tumor samples and 100 healthy breast tissues of GSE88883 through unpaired *t*-test, which resulted in 13,575 CpGs that were still significantly hyper-methylated in tumor samples. The top 200 most significant ones according to their FDR adjusted *P* values were used to hierarchical clustering analysis, through which the normal tissue samples could be distinctly separated from those tumor tissue samples as shown in Figure 4B. While blood samples from 51 BRCA patients and 41 healthy person (GSE89093) [37] were mixed up within the normal tissue group, which indicated the tissue specificity of methylation. Eight CpGs, including cg02156680, cg05227549, cg06100368, cg16171526, cg18565473, cg22548054, cg25097074, and cg25199322, were finally retained out of the 13,575 CpGs and used as BRCA-specific diagnostic markers for their significantly higher methylations in BRCA tumor samples than any of the other 10 cancers’ tumor as well as adjacent normal samples. Left and right panel of Figure 4C shows the adjacent normal and tumor samples’ mean methylation level of the eight BRCA-specific diagnosis CpGs in BRCA and other ten cancers, respectively.

### CpG-based BRCA-specific diagnostic model evaluation

**Figure 5:**
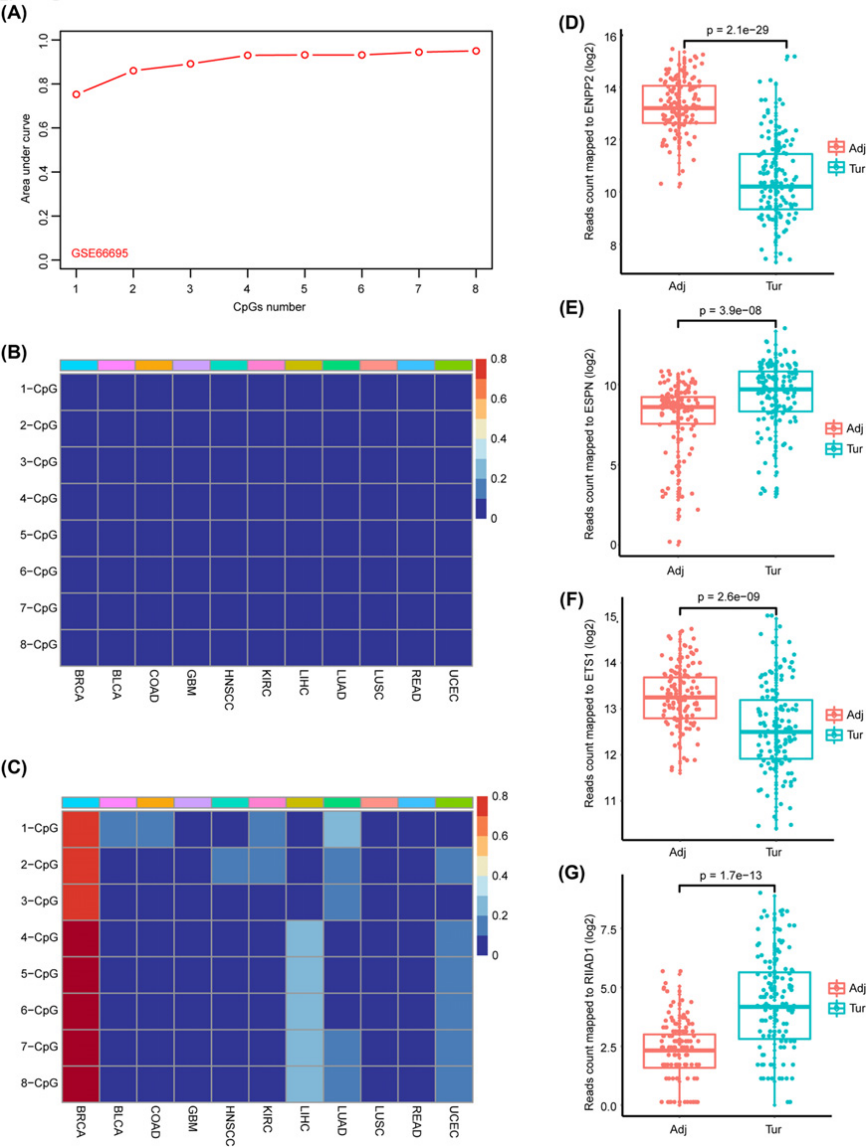
Evaluation of the BRCA-specific diagnostic model (**A**) AUC for evaluating performance of the logistic regression model in distinguishing BRCA and normal samples when progressively including CpGs according to their ranking in information-gain-based analysis. (**B**) Heatmap shows percent of normal control samples of GSE66695 and GSE89093 from GEO and adjacent normal samples of other ten cancers from TCGA that included as BRCA by the logistic regression model when progressively including the eight CpGs. (**C**) Heatmap shows percent of tumor samples of other ten cancers from TCGA that included as BRCA by the logistic regression model when progressively including the eight CpGs. (**D–G**) Boxplots for expression values represented by log2-based counts of mapping reads of four genes, including ENPP2, ESPN, ETS1 and RIIAD1, that containing six of the eight BRCA-specific diagnostic CpGs.

We used information gain-based feature selection method, a machine learning algorithm, to rank the eight BRCA-specific CpGs. Logistic regression models were trained based on the TCGA-BRCA dataset by using sample type, i.e. tumor and normal, as categorical responsive variables, and including β values of one to eight CpGs in the order of their rankings, as continuous predictive variables. The AUC could achieve 0.86 in the testing set GSE66695 when only included two CpGs in the logistic regression model and 0.93 when four CpGs were included, after which the AUC increased very slowly with more CpGs included as shown in Figure 5A. So, we concluded that combination of four CpGs should be more appropriate taking both accuracy and cost-effective into account. Figure 5B,C respectively illustrated percentage of normal and tumor samples of other 10 common cancers in TCGA that were defined as BRCA by the logistic regression model when including one to eight CpGs.

The eight BRCA-specific CpGs were annotated to four genes: cg02156680 (ENPP2), cg18565473 (ETS1), cg25097074 (ESPN), cg06100368, and cg25199322 (RIIAD1). Cg05227549, cg16171526, and cg22548054 were not annotated by any gene. We investigated correlations between methylation levels of each pair CpGs that located at the promoter regions of the four genes. As a result, most CpG pairs exhibited significantly positive correlations (Supplementary Figure S2), which were consistent that nearby CpGs tend to be highly co-methylation [38]. Besides, expressions of ENPP2 and ETS1 were significantly decreased in BRCA tumor samples, and ESPN and RIIAD1 were significantly elevated in BRCA tumor samples compared with adjacent normal samples (Figure 5D–G). Expressions of those four genes in MCF-7 and MDA-MB-231 cell lines, representing luminal A and triple negative breast cancer cell lines, respectively, were also explored in another independent public dataset (GSE3156) [39]. As a result, consistent with their expression changes in tumor samples compared with adjacent samples, ENPP2 and ETS1 showed decreased expression levels in MDA-MB-231, a highly aggressive cell line, compared with MCF-7, a weakly aggressive cell line. And ESPN and RIIAD1 illustrated increased expression levels in MDA-MB-231 compared with MCF-7 cell line (Supplementary Figure S3). Those should imply potential roles of those four genes in BRCA initiation and progression.

### Chromatin accessible landscape of BRCA-specific CpGs annotated genes

**Figure 6:**
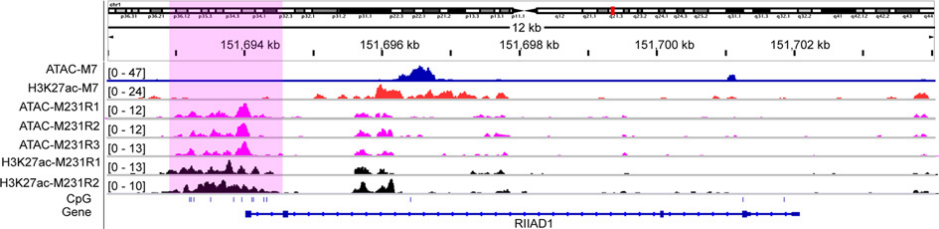
Chromatin accessibility states of RIIAD1 in MCF-7 and MDA-MB-231 cell lines reflected by ATAC-seq H3K27ac as an active enhancer marker whose abundances at RIIAD1 in MCF-7 and MDA-MB-231 cell lines were also provided as the corresponding ChIP-seq reads intensity. Locations of CpGs across RIIAD1 were indicated in the eighth track with short vertical lines. Pink shadow highlights promoter region showing different chromatin accessibility states between MCF-7 and MDA-MB-231 and enriched with CpGs.

Aberrant DNA methylation may contribute changed chromatin accessibility that could consequently cause alteration in gene transcription if the affected region is happened to be CRE. In the present study, we explored accessibility landscapes of ENPP2, ETS1, ESPN, and RIIAD1 through ATAC-seq datasets in MCF-7 and MDA-MB-231 cell lines. As a result, RIIAD1 was more accessible in promoter and enhancer region (marked by enriched H3K27ac ChIP-seq reads) that enriched with CpGs, i.e. CGI, in MDA-MB-231 cell lines than that in MCF-7 cell lines as shown in Figure 6, which was consistent with its elevated expression in tumor samples. Similar relations between CpGs and gene accessibilities of ETS1, ESPN, and ENPP2 were also obtained and illustrated in Supplementary Figures S4–6.

## Discussion

We here reported a comprehensive analysis of DNAme and gene expression profiles of BRCA and other 10 common cancers, through which 8 BRCA-specific CpGs that could sensitively and specifically distinguish BRCA from normal samples, as well as other cancers, were identified. Mechanistic exploration implies potential manipulation of CpGs in chromatin accessibility, by which aberrant methylation may modulate expression of cancer-related genes.

Cancer early screening and diagnosis is a worldwide puzzled problem that we have been focusing on. Imaging techniques represent the most broadly used and relative mature means, but their disadvantages are also obvious, e.g. expensive. Besides, imaging techniques could barely be applied in cancer early screening. Cancer screening is included in most of health checkup by detecting serum tumor biomarkers, including CA72-4, CA19-9, and so on, but the sensitivity and specificity are both unsatisfactory for its broad spectrum, as well as personal difference in biomarkers’ response [40,41]. So, identification of cancer type-specific biomarkers is urgently needed for clinical decision making.

Popularization of high-throughput gene sequencing or microarray techniques have greatly accelerated the identification of disease-specific diagnosis or treatment targets in genome-wide scale. Global DNA methylation profiling could be readily achieved by methylation microarray and sequencing, through which comparisons of methylation levels could be performed between different status, such as cancer versus normal, for identify valuable status signatures. Lots of methylation-based diagnostic and prognostic biomarkers for cancer have been increasingly developed, and CIMP represents one of the most popular ones since its first proposal in colorectal cancer. In the present study, BRCA samples with CIMP showed significantly inferior OS compared with those without CIMP, which was consistent with observations in lots of other cancers [42,43]. Fang et al. [44] also performed CIMP analysis among 39 BRCA patients with similar method of the present study, i.e. consensus clustering analysis based on the most variable CpGs, and defined those patients that with coincident cancer-specific hypermethylation at some CpGs as having CIMP. But contrary conclusions were drawn in Fang’s study, i.e. patients with CIMP tend to have superior overall survival and low metastasis risk compared with those without CIMP, which might be caused by differences in feature selection for CIMP definition. So, unification is still needed for CIMP identification in specific cancer before its clinical application for patient stratification.

Speaking of BRCA diagnosis, multiple DNA methylation makers have been previously reported. Explorations of possibilities of application of blood-based, including whole blood and cell-free DNA (cfDNA) from serum and plasma, methylation markers in BRCA screening have been conducted for its noninvasion and convenience in sampling [45]. While, there are also some remarkable limitations in blood-based method. For example, biased selection of studied targets in gene-specific level methylation measurement through methylation-specific PCR or pyrosequencing, in particular, BRCA1 in whole blood [46,47] and RASSF1A in cfDNA [48–51], and heterogeneous conclusions drawn from different epigenome-wide studies [45]. Besides, consistency of methylation levels between DNA from tumor tissues and blood samples, particular those cfDNA, are yet to be confirmed. Lots of studies were focused on differences of gene-specific or epigenome-wide DNA methylations between BRCA and normal tissue samples for identifying BRCA diagnostic markers. Siqurdsson et al. [52] identified promoter methylation of ALKBH3 as an important BRCA marker through comprehensively analyzing BRCA methylation microarray datasets from GEO and TCGA, and pyrosequencing in BRCA and normal tissue samples. Diagnostic value of elevated DUSP1 promoter methylation in peripheral blood and tumor tissue for triple negative breast cancer was strongly supported by Pang et al [53]. In the present study, we systematically compared overall DNA methylation profiles of BRCA samples to not only adjacent and normal breast tissues, but also other 10 common cancers, and constructed a logistic regression model through which BRCA could be distinguished from other sample types with high sensitivity and specificity. Shi et al. [54] conducted a pan-cancer DNA methylation analysis and developed a seven CpG-based model for classifying pan-cancer and normal tissue, and a 12 CpG-based model for classifying different cancer types. Compared with Shi’s study, our model could achieve a good performance when only included four CpGs and should be more cost-effective.

A total of four genes including ENPP2, ESPN, ETS1, and RIIAD1 were annotated by the eight CpGs. Functions of all those four genes but RIIAD1 have been extensively studied. ETS1 is a well-known proto-oncogene, which encode a transcription factor that belongs to ETS family, that biologically involves in stem cell development, cell senescence and death, and tumorigenesis. ETS1 is conventionally recognized as an oncogene in most of cancers [55,56], which is conflicted with the result of our study that illustrates significantly decreased ETS1 mRNA levels in BRCA tumor samples compared with adjacent normal samples (Figure 5F for details). So, we speculated that ETS1 might phenotypically act different among multiple cancers. To this end, we compared the mRNA levels of ETS1 between tumor and normal tissues of all cancers deposited in TCGA by using Gene Expression Profiling Interactive Analysis (GEPIA, http://gepia.cancer-pku.cn/). Strikingly, ETS1 is significantly down-regulated in all the gynecologic cancers, including BRCA, cervical squamous cell carcinoma and endocervical, ovarian serous cystadenocarcinoma, uterine corpus endometrial carcinoma, and uterine carcinosarcoma, out of the 33 cancers (Supplementary Figure S7). Besides, survival analysis through kmplotter online tool (http://kmplot.com/analysis/) indicated the favorable role of high ETS1 mRNA level for BRCA relapse-free survival (Supplementary Figure S7). So, ETS1 might be a novel tumor suppressor for BRCA, or more generally for gynecologic cancers, that is well worth for future experimental and clinical research. ENPP2 encodes a protein that biologically contributes both the stripping of phosphodiester from DNA and catalyzation of lysophosphatidic acid production. It can functionally promote tumor cells’ motility and angiogenic. Elevated ENPP2 expression has been previously reported in several carcinomas, such as liver cancer [57], and studies about ENPP2 in breast cancer are all cell-based [58,59]. Decreased ENPP2 mRNA levels in tumor tissues compared with adjacent normal tissues of several gynecologic cancers including cervical squamous cell carcinoma and endocervical, uterine corpus endometrial carcinoma, and uterine carcinosarcoma in addition to BRCA were observed through GEPIA (data not shown). Besides, association between high ENPP2 mRNA level and superior BRCA relapse-free survival was also confirmed using kmplotter (data not shown). So, ENPP2 might be a potential biomarker in BRCA initiation and prognosis. There has no study for association between ESPN or RIIAD1 and BRCA, while, their aberrant mRNA expressions, as well as alterations of methylations of their CpGs should reveal their potential roles in BRCA.

Dynamic of chromatin accessibility is closely associated with natural cells’ functions as well as pathological processes, and is regulated by both internal developmental cues and external stimulates, such as competition between transcription factor and histone. While association between DNA methylation and chromatin accessibility was rarely reported. In Peres’s study [60], it was reported that chromatin accessibility along with G-quadruplex could extensively influence CpG island methylation, but the effect of CpG methylation on chromatin accessibility was not studied. Smale et al. [29] described a class of primary response gene that could be activated without new protein synthesis and characterized by CpG island promoter, whose promoter chromatin could be directly loosed via the occupy of CpGs at unstable nucleosomes independent of SWI/SNF nucleosome remodeling complex. In our study, widely divergent accessibilities in CpG island promoters/enhancers of BRCA-specific CpGs annotated genes were observed between breast cancer cell lines with different invasiveness, i.e. MCF-7 and MDA-MB-231. We hypothesized that different CpG island methylation levels may partly influence the occupy process of CpG island at unstable nucleosomes that described by Smale et al., which ultimately result in aberrant gene expression as our result (Figure 5D–G).

Limitations also exist in the present study. Samples used in the present study were tissues, which may make it difficult to implicate our diagnostic model in BRCA early screening. Validation of methylation levels’ differences of the eight BRCA-specific CpGs between BRCA and normal samples was not included in the present study and would be conducted in future.

In conclusion, we obtained a diagnostic model through which BRCA could be distinguished from both normal and other ten common cancer samples. BRCA-specific CpGs annotated genes may be associated with BRCA initiation and progression. In addition, we also preliminarily identify the potential association between aberrant CpG methylation and the architecture of surrounding DNA for the first time. Our study should shed some new light on the underlying mechanism of progression as well as clinical diagnosis research of BRCA.

## Supplementary Material

Supplementary Figures S1-S7

## Data Availability

These data were derived from the following resources available in the public domain: https://portal.gdc.cancer.gov/ https://www.ncbi.nlm.nih.gov/geo/query/acc.cgi?acc=GSE88883 https://www.ncbi.nlm.nih.gov/geo/query/acc.cgi?acc=GSE66695 https://www.ncbi.nlm.nih.gov/geo/query/acc.cgi?acc=GSE89093 https://www.ncbi.nlm.nih.gov/geo/query/acc.cgi?acc=GSE101736 https://www.ncbi.nlm.nih.gov/geo/query/acc.cgi?acc=GSE89093 https://www.ncbi.nlm.nih.gov/geo/query/acc.cgi?acc=GSE78113 https://www.ncbi.nlm.nih.gov/geo/query/acc.cgi?acc=GSE72141 https://www.ncbi.nlm.nih.gov/geo/query/acc.cgi?acc=GSE3156

## Abbreviations

BRCA: breast cancer
cfDNA: cell-free DNA
CGI: CpG island
CIMP: CpG island methylator phenotype
CRE: cis-regulation element
DDR: DNA damage repair
GEO: Gene Expression Omnibus
K-S: Kolmogorov–Smirnov
TCGA: the Cancer Genome Atlas
