# Supplementary material for: Integrative analysis of DNA methylation and gene expression profiles identified potential breast cancer-specific diagnostic markers
Source: Biosci Rep. 2020 May 27;40(5):BSR20201053. doi: 10.1042/BSR20201053 (PMC7263199; doi:10.1042/BSR20201053)
Supplement: Supplementary Figures S1-S7 [file BSR-2020-1053_supp.pdf]

Figure S1

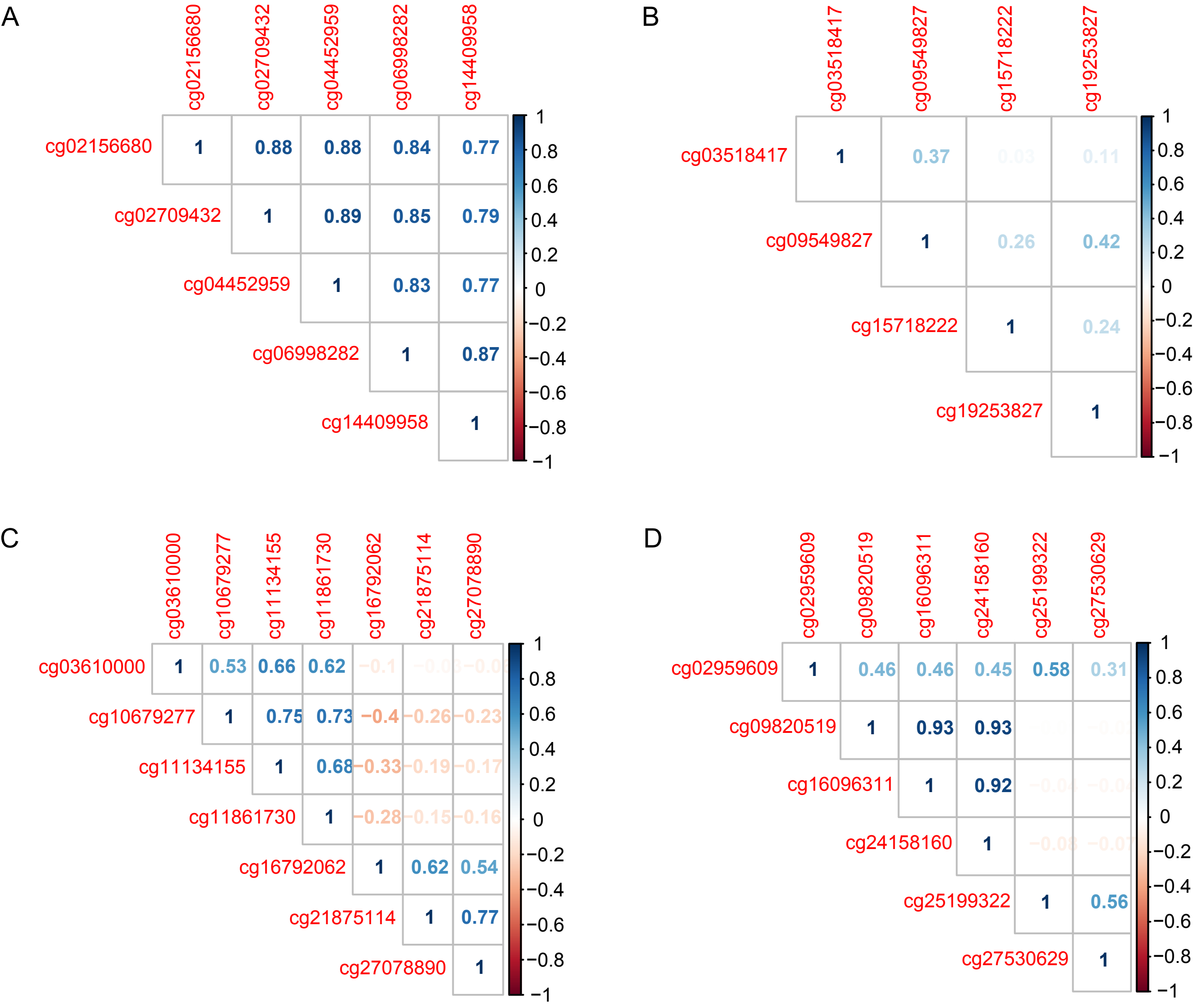
A
cg02156680
cg02709432
cg04452959
cg06998282
cg14409958
1 0.88 0.88 0.84 0.77
1 0.89 0.85 0.79
1 0.83 0.77
1 0.87
1
B
cg03518417
cg09549827
cg15718222
cg19253827
1 0.37 0.03 0.11
1 0.26 0.42
1 0.24
1
C
cg03610000
cg10679277
cg11134155
cg11861730
cg16792062
cg21875114
cg27078890
1 0.53 0.66 0.62 −0.1
1 0.75 0.73 −0.4 −0.26 −0.23
1 0.68 −0.33 −0.19 −0.17
1 −0.28 −0.15 −0.16
1 0.62 0.54
1 0.77
1
D
cg02959609
cg09820519
cg16096311
cg24158160
cg25199322
cg27530629
1 0.46 0.46 0.45 0.58 0.31
1 0.93 0.93
1 0.92
1
1 0.56
1
1 0.8 0.6 0.4 0.2 0 −0.2 −0.4 −0.6 −0.8 −1

Figure S1 Consensus clustering analysis of all the BRCA tumor samples.

Figure S2

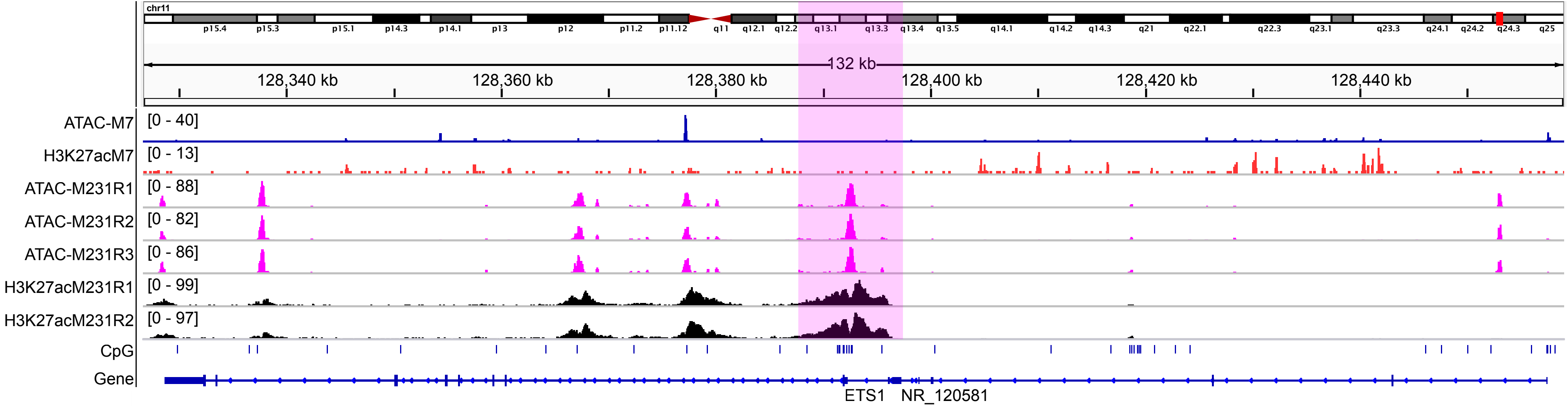
chr11
p15.4
p15.3
p15.1
p14.3
p14.1
p13
p12
p11.2
p11.12
q11
q12.1
q12.2
q13.1
q13.3
q13.4
q13.5
q14.1
q14.2
q14.3
q21
q22.1
q22.3
q23.1
q23.3
q24.1
q24.2
q24.3
q25
132 kb
128,340 kb
128,360 kb
128,380 kb
128,400 kb
128,420 kb
128,440 kb
ATAC-M7
[0 - 40]
H3K27acM7
[0 - 13]
ATAC-M231R1
[0 - 88]
ATAC-M231R2
[0 - 82]
ATAC-M231R3
[0 - 86]
H3K27acM231R1
[0 - 99]
H3K27acM231R2
[0 - 97]
CpG
Gene
ETS1
NR_120581

Figure S2 Upper triangular heatmap illustrates pearson correlations between any two CpGs that located in the promoter of ENPP2 (A), ESPN (B), ETS1 (C) and RIIAD1 (D). Color bar on the right of every heatmap shows the gradient correlation coefficients.

Figure S3

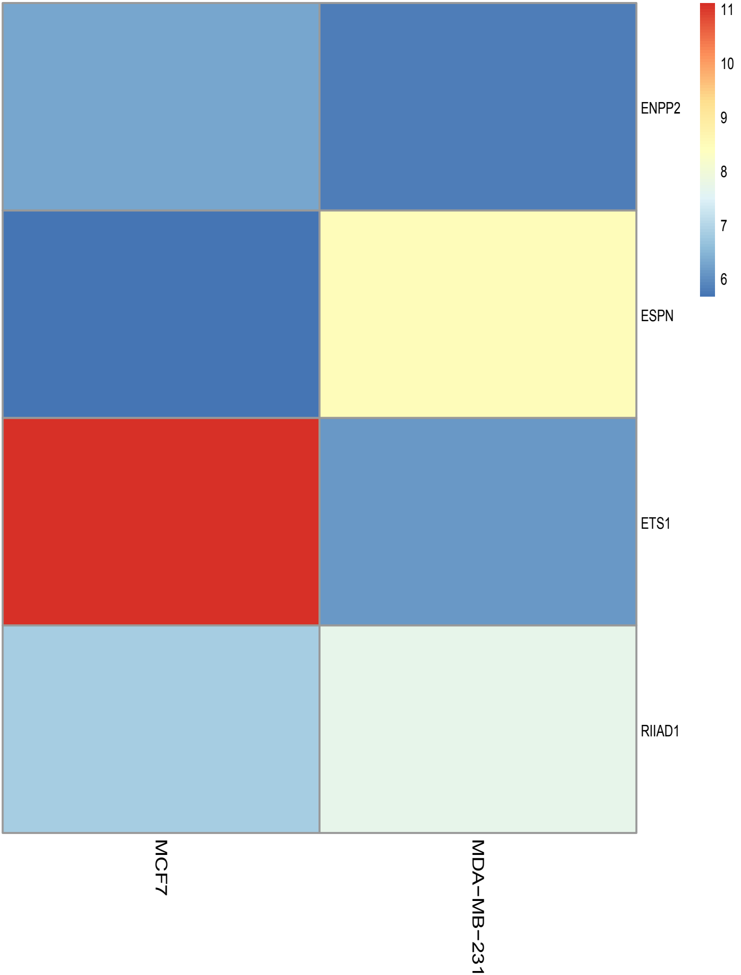

Figure S3 Heatmap illustrating mRNA levels of ENPP2, ESPN, ETS1, and RIIAD1 in MCF-7 and MDA-MB-231 cell lines based on GSE3156 dataset.

Figure S4

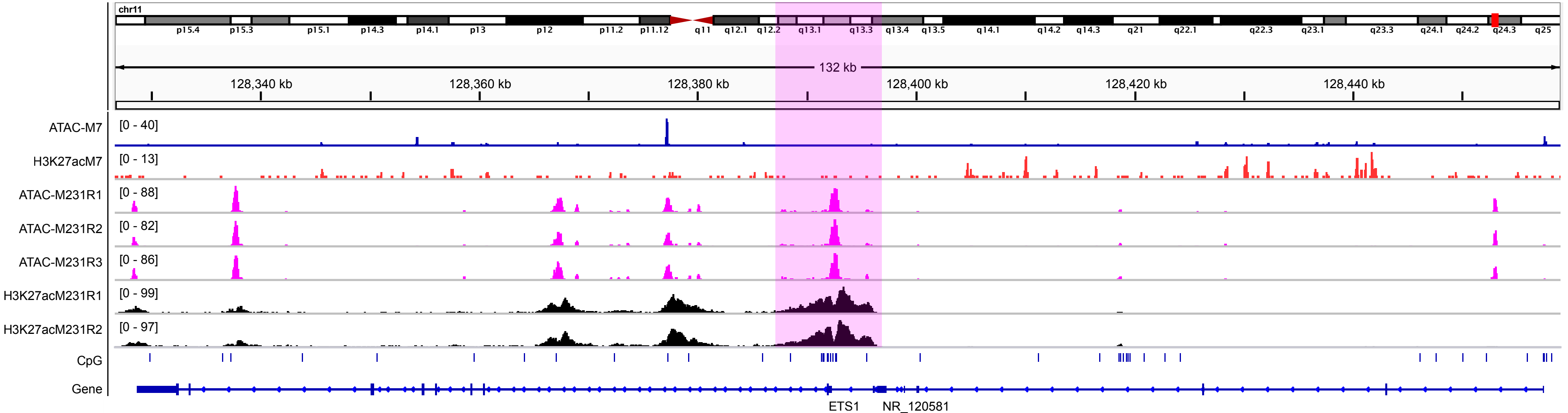
chr11
p15.4
p15.3
p15.1
p14.3
p14.1
p13
p12
p11.2
p11.12
q11
q12.1
q12.2
q13.1
q13.3
q13.4
q13.5
q14.1
q14.2
q14.3
q21
q22.1
q22.3
q23.1
q23.3
q24.1
q24.2
q24.3
q25
132 kb
128,340 kb
128,360 kb
128,380 kb
128,400 kb
128,420 kb
128,440 kb
ATAC-M7
[0 - 40]
H3K27acM7
[0 - 13]
ATAC-M231R1
[0 - 88]
ATAC-M231R2
[0 - 82]
ATAC-M231R3
[0 - 86]
H3K27acM231R1
[0 - 99]
H3K27acM231R2
[0 - 97]
CpG
Gene
ETS1
NR_120581

Figure S4 Chromatin accessibility states of ETS1 in MCF-7 and MDA-MB-231 cell lines.

Figure S5

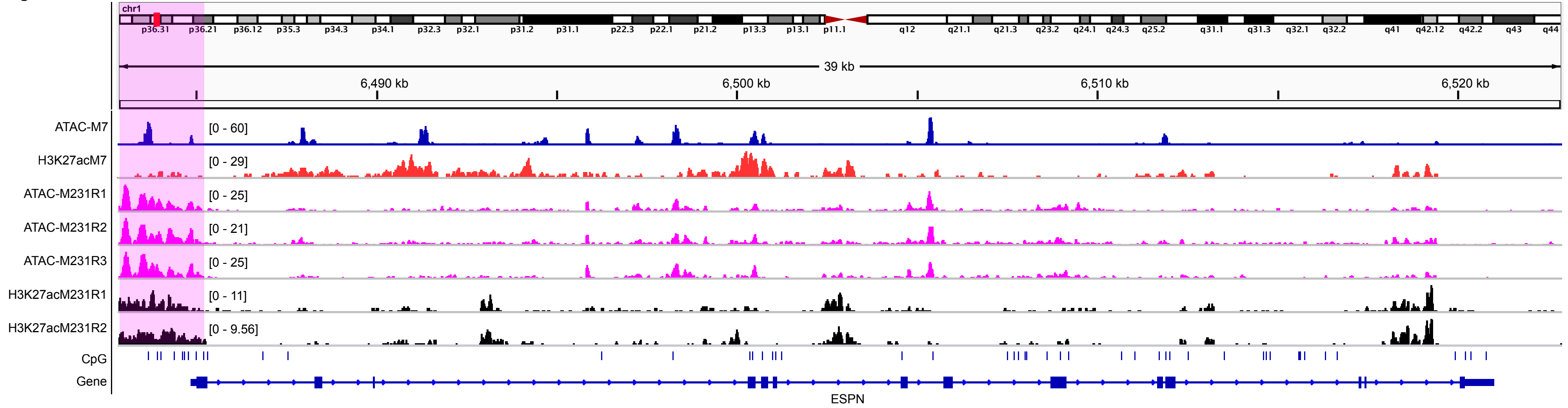
chr1
p36.31
p36.21
p36.12
p35.3
p34.3
p34.1
p32.3
p32.1
p31.2
p31.1
p22.3
p22.1
p21.2
p13.3
p13.1
p11.1
q12
q21.1
q21.3
q23.2
q24.1
q24.3
q25.2
q31.1
q31.3
q32.1
q32.2
q41
q42.12
q42.2
q43
q44
39 kb
6,490 kb
6,500 kb
6,510 kb
6,520 kb
ATAC-M7
[0 - 60]
H3K27acM7
[0 - 29]
ATAC-M231R1
[0 - 25]
ATAC-M231R2
[0 - 21]
ATAC-M231R3
[0 - 25]
H3K27acM231R1
[0 - 11]
H3K27acM231R2
[0 - 9.56]
CpG
Gene
ESPN

Figure S5 Chromatin accessibility states of ESPN in MCF-7 and MDA-MB-231 cell lines.

Figure S6

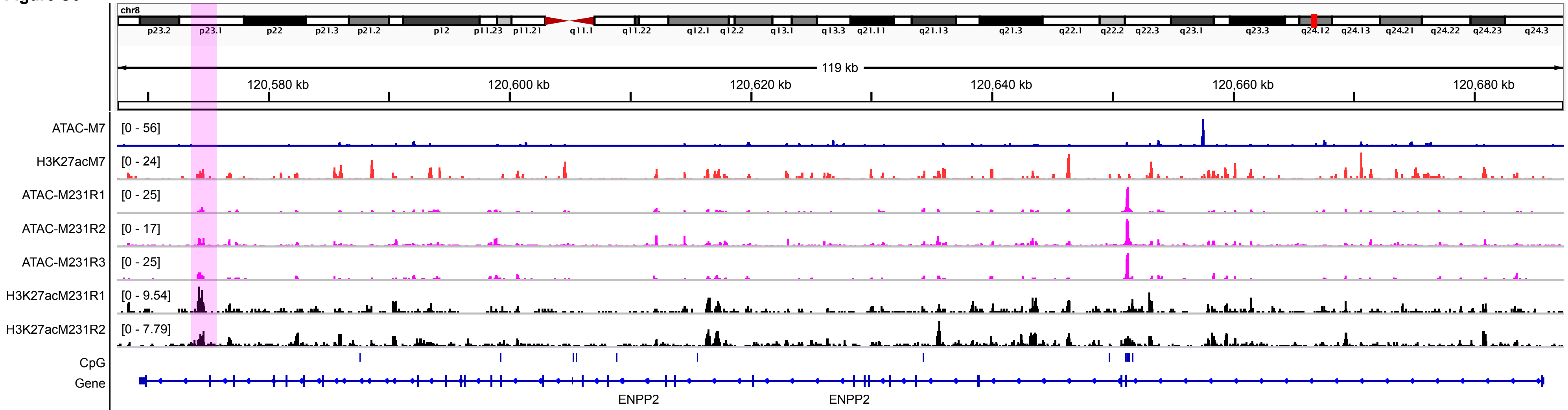

chr8
p23.2
p23.1
p22
p21.3
p21.2
p12
p11.23
p11.21
q11.1
q11.22
q12.1
q12.2
q13.1
q13.3
q21.11
q21.13
q21.3
q22.1
q22.2
q22.3
q23.1
q23.3
q24.12
q24.13
q24.21
q24.22
q24.23
q24.3
119 kb
120,580 kb
120,600 kb
120,620 kb
120,640 kb
120,660 kb
120,680 kb
ATAC-M7
[0 - 56]
H3K27acM7
[0 - 24]
ATAC-M231R1
[0 - 25]
ATAC-M231R2
[0 - 17]
ATAC-M231R3
[0 - 25]
H3K27acM231R1
[0 - 9.54]
H3K27acM231R2
[0 - 7.79]
CpG
Gene
ENPP2
ENPP2

Figure S6 Chromatin accessibility states of ENPP2 in MCF-7 and MDA-MB-231 cell lines.

Figure S7

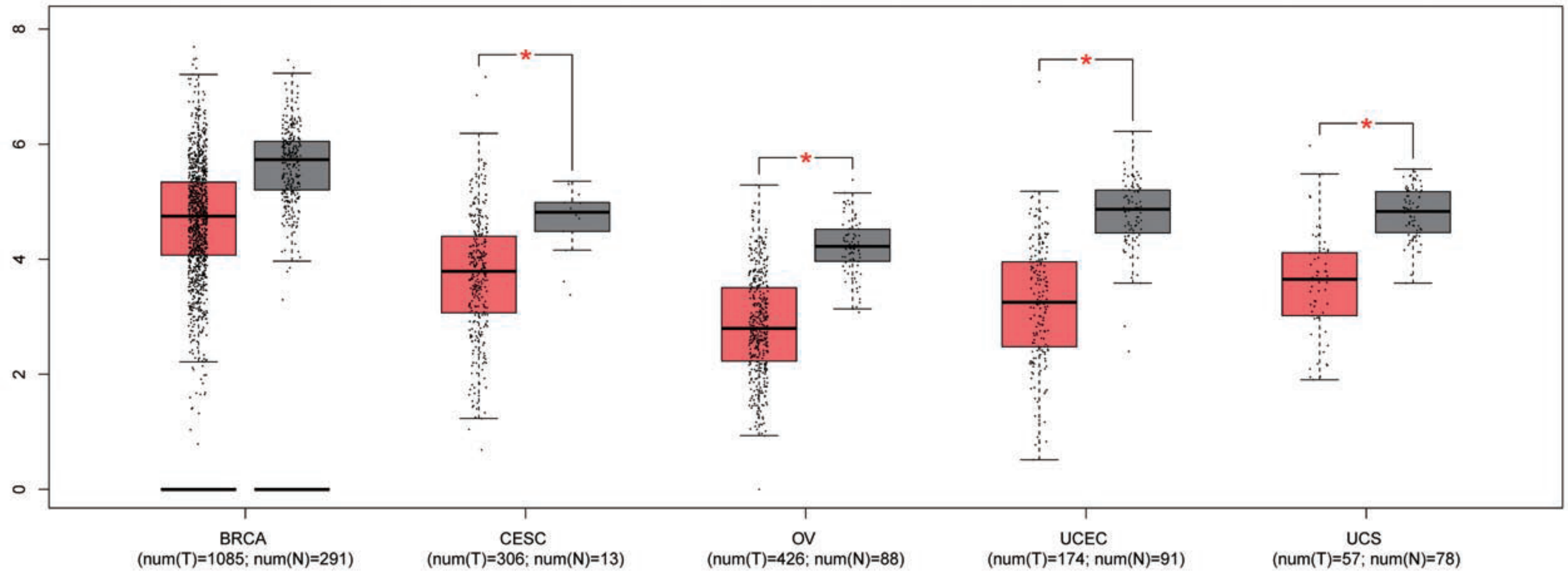

8
6
4
2
0
*
*
*
*
BRCA
(num(T)=1085; num(N)=291)
CESC
(num(T)=306; num(N)=13)
OV
(num(T)=426; num(N)=88)
UCEC
(num(T)=174; num(N)=91)
UCS
(num(T)=57; num(N)=78)

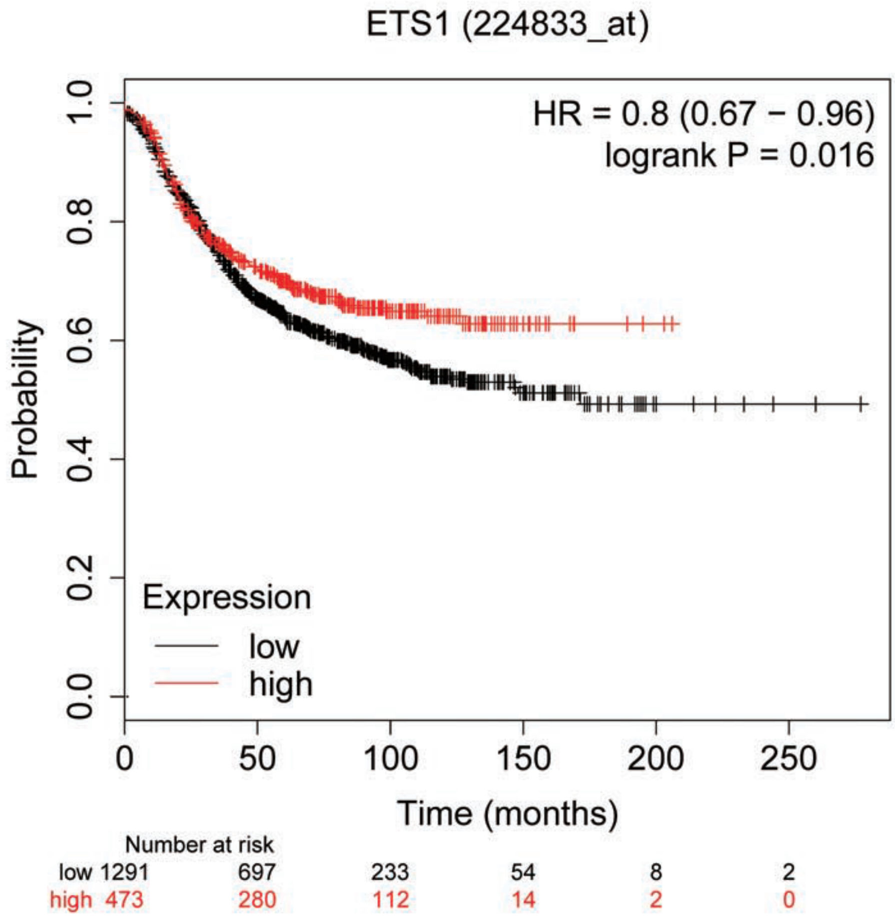

ETS1 (224833_at)
HR = 0.8 (0.67 − 0.96)
logrank P = 0.016
Probability
0.0
0.2
0.4
0.6
0.8
1.0
Expression
low
high
0
50
100
150
200
250
Time (months)
Number at risk
low 1291 697 233 54 8 2
high 473 280 112 14 2 0

Figure S7 ETS1 is down-regulated in all gynecological cancer out of the 33 cancers in TCGA and has inferior prognostic value in BRCA.
